# Self-Expanding Metal Stents in the Acute Management of Oesophageal Variceal Bleeding: A Systematic Review

**DOI:** 10.7759/cureus.73517

**Published:** 2024-11-12

**Authors:** Eoghan Burke, Patricia Harkins, Mayilone Arumugasamy

**Affiliations:** 1 Surgery, Royal College of Surgeons in Ireland, Dublin, IRL; 2 Medicine, Royal College of Physicians in Ireland, Dublin, IRL

**Keywords:** balloon tamponade, danis stent, liver failure, meld score, variceal bleeding

## Abstract

Acute variceal bleeding (AVB) continues to challenge physicians and healthcare systems. Despite significant advances in our multimodal approach to managing this problem, namely medical, endoscopic, and radiological techniques, the mortality rates for this patient cohort remain as high as 20% on the index admission. This mortality rate has remained unchanged over the past 25 years. A crucial tool in the management of AVB is the balloon tamponade technique. However, this is associated with numerous severe and potentially life-threatening adverse events.

Due to the limitations of oesophageal balloon tamponade devices, there has been an increased interest in using self-expanding metal stents (SEMS) to manage refractory variceal bleeding. There is a base of experience in using SEMS derived from their use in managing malignant obstructions.

This study aimed to synthesise all available evidence, for the first time, on using SEMS to manage refractory oesophageal AVB. This study was a systematic review of published papers, which is reported per the Preferred Reporting Items for Systematic Reviews and Meta-Analyses (PRISMA) statement.

We identified 16 suitable studies for review. These comprised one randomised controlled trial comparing SEMS to balloon tamponade, one prospective cohort study, nine retrospective cohort studies, four case reports, and one case series. In total, 246 patients were included.

An average survival rate of 49% was seen among the 11 studies that reported a six-week survival rate; this included a cohort of 225 patients. The SEMS were left in situ for an average of 7.5 days, with a maximum average of 18 days in one study. The average rate for controlling the acute bleeding episode in patients receiving a SEMS was 96%. The re-bleeding rate on the removal of SEMS was 5%, far superior to the widely reported 50% re-bleeding rate for the balloon tamponade technique. The adverse event profile for the SEMS appears superior to the balloon tamponade technique overall. The rate of stent-related adverse events in patients receiving a SEMS was 25%. The most common adverse events were technical issues related to stent functioning, namely stent migration. The most severe stent-related adverse event was compression of the left main bronchus in two cases, which required the removal of the stent.

Our study has several limitations, which we have alluded to throughout the paper. The studies on this issue are of poor quality, with only one randomised controlled trial performed. As a result, we must interpret the results of our research with caution.

Our study supports the use of SEMS in managing AVB as a promising area of research. We have highlighted that further well-designed randomised controlled trials are needed to assess the efficacy of this technique, ideally compared directly to the balloon tamponade technique. However, based on this systematic review, the current body of evidence would suggest that the SEMS is superior to the balloon tamponade technique in terms of adverse event profile, re-bleeding rate, and length of time the device can safely be left in situ. Current evidence suggests that SEMS are as effective at controlling acute bleeding episodes as the balloon tamponade technique.

## Introduction and background

Acute variceal bleeding (AVB) continues to challenge physicians and healthcare systems. Despite significant advances in our multimodal approach to managing this problem, namely medical, endoscopic, and radiological techniques, the mortality rates for this patient cohort remain as high as 20% on the index admission. This mortality rate has remained unchanged over the past 25 years [1].

Current guidelines suggest using vasoactive medications such as terlipressin and endoscopy as the initial treatments of choice [2]. For oesophageal varices, the preferred endoscopic haemostatic technique is variceal band ligation [3].

Current guidelines for re-bleeding, defined by the Baveno consensus group as a clinically significant episode of melena or haematemesis occurring within five days of the initial treatment [4], support using a second attempt at endoscopic management. However, when endoscopic and maximal medical therapy fails, the next optimal option is to pursue a transjugular intrahepatic portosystemic shunt (TIPSS). This procedure is usually available solely in specialist centres, precluding its immediate use in many patients.

Classically, as a temporising measure, the use of a balloon tamponade device, most commonly the Sengstaken Blakemore tube [5], has been implemented to bridge the gap to definitive TIPSS [6]. Despite its efficacy in controlling acute bleeding, with rates reported as high as 90% [7], the balloon tamponade technique is associated with a plethora of potentially life-threatening issues. Indeed, it has been reported that life-threatening complications arise in 20%-60% of cases where these tubes are used [8].

As the tubes are designed to tamponade both the gastro-oesophageal junction and the oesophagus, they render the patient dysphagic, so much so that the patient is not able to swallow their saliva. This accounts for some of the more common complications of these tubes being cases of aspiration pneumonia [8]. More serious adverse events include oesophageal perforation and ischaemic necrosis of the mucosa, which is why these tubes cannot be left in situ for over 24 hours. Malnutrition in cirrhotic patients suffering a variceal bleed is known to be a poor prognostic indicator, and patients being managed with a balloon tamponade device cannot receive enteral nutrition [9].

Due to the limitations of oesophageal balloon tamponade devices, there has been an increased interest in using self-expanding metal stents (SEMS) to manage refractory variceal bleeding. There is a base of experience in using SEMS derived from their use in managing malignant obstructions [10].

Indeed, the most recent Baveno 7 consensus meeting on portal hypertension, held in October 2021, suggested that SEMS may be used instead of balloon tamponade devices in managing refractory variceal bleeding [11]. However, evidence for their use is currently weak. As such, we aimed to assess the available evidence to date on using SEMS in managing refractory AVB to guide future research into this promising area.

## Review

Materials and methods

Study Aims and Objectives

This study aimed to synthesise available evidence on using SEMS to manage refractory oesophageal AVB. We utilised the Baveno consensus guidelines to determine our primary and secondary outcomes.

Primary Outcomes

As suggested by the Baveno consensus group, the primary outcome for any study evaluating the management of either a first or refractory episode of AVB should be six-week mortality. We set this as our primary outcome.

Secondary Outcomes

Our secondary outcomes included rate of control of the acute bleeding episode (<5 days), re-bleeding rates following removal of the SEMS, details on further treatment following SEMS placement, transfusion requirement, length of time the SEMS was left in situ, and adverse events.

To better inform our search strategy, we formalised our search question using the population, intervention, comparison, and outcomes (PICO) model (Table 1).

**Table 1:** PICO tool used to develop the studies search string PICO: population, intervention, comparison, and outcomes

P	I	C	O
Population	Intervention	Comparison	Outcome
Adult patients presenting with an acute oesophageal variceal bleed.	Self-expanding metal stent.	Either no comparator or a balloon tamponade device.	Ideally should report on the primary outcome listed above as well as the secondary outcomes listed above.

Study Design

This study was a systematic review of published papers, which is reported per the Preferred Reporting Items for Systematic Reviews and Meta-Analyses (PRISMA) statement [12]. The study protocol was registered prospectively in the International Prospective Register of Systematic Reviews (PROSPERO).

Eligibility Criteria

Inclusion criteria for this study included randomised controlled trials, observational studies (both prospective and retrospective), and case reports. The decision to include case reports was conscious, as, given the emergent presentation of these patients, it is difficult to conduct randomised controlled trials, so case reports can offer valuable insights. The included studies should report on adult patients presenting with an acute oesophageal variceal bleed. The included studies should assess the performance of a self-expanding metal stent in the acute management of a variceal bleeding episode. Studies reporting on the primary outcome were considered for inclusion.

Studies involving paediatric patients were not included in this study.

Search Strategy

A detailed search strategy was developed in conjunction with a medical librarian. Keywords and Medical Subject Headings (MeSH) terms relating to variceal bleeding and self-expanding metal stents were used to create the search string: (oesophagal stent) OR (Oesophageal stent)) AND ((sengstaken Blakemore) OR (Oesophageal balloon tamponade) OR (oesophagal balloon tamponade) OR (sengstaken Blakemore Tube))) AND ((Variceal bleeding) OR (Upper GI Bleeding) OR (Varices))).

This search string was then applied to the following bibliographic databases: PubMed, Excerpta Medica database (EMBASE), and the Cochrane Central Register of Controlled Trials (CENTRAL). We used Google Scholar to conduct a citation search, described below, during the study selection process. This ensured that we were unlikely to miss relevant studies while limiting the number of initial results to be screened. All databases were searched from inception. The databases were interrogated from June 30^th^, 2024, to 1^st^ August 2024.

Rayyan software (Rayyan Systems Inc., Cambridge, MA), a systematic review management software package was used during this systematic review [13].

Study Selection

After removing duplicates, two authors independently screened all identified studies’ titles and abstracts. Abstracts meeting the previously described inclusion criteria were selected. If there was any conflict about a study’s inclusion, this was resolved by a third author. The resulting studies were reviewed in full, and eligibility for inclusion in qualitative and quantitative analysis was determined. Any conflict about a study’s eligibility was resolved with consensus. During the complete article review, a hand search of references was conducted to identify any studies not identified in the original search. Similarly, a citation search using Google Scholar on all eligible articles was completed again to ensure no further studies were omitted.

Data Extraction

Two authors independently extracted data from the selected studies using a predetermined data extraction form developed using Microsoft Excel (Microsoft Corp., Redmond, WA). The data extracted included the authors, study design, year of publication, participant demographics, Model for End-Stage Liver Disease (MELD) score, and primary and secondary outcomes, as previously listed.

Risk of Bias Assessment

Two authors independently assessed the included studies’ risk of bias using validated tools. Any conflicts about a study's risk of bias were resolved initially by consensus and, if needed, by a third author. The Cochrane Collaboration’s risk of bias tool was used for randomised controlled trials [14]. For cohort studies, the JBI’s cohort studies checklist was used [15], and the JBI’s case report checklist was used for case reports [15].

Results

Study Selection

**Figure 1:**
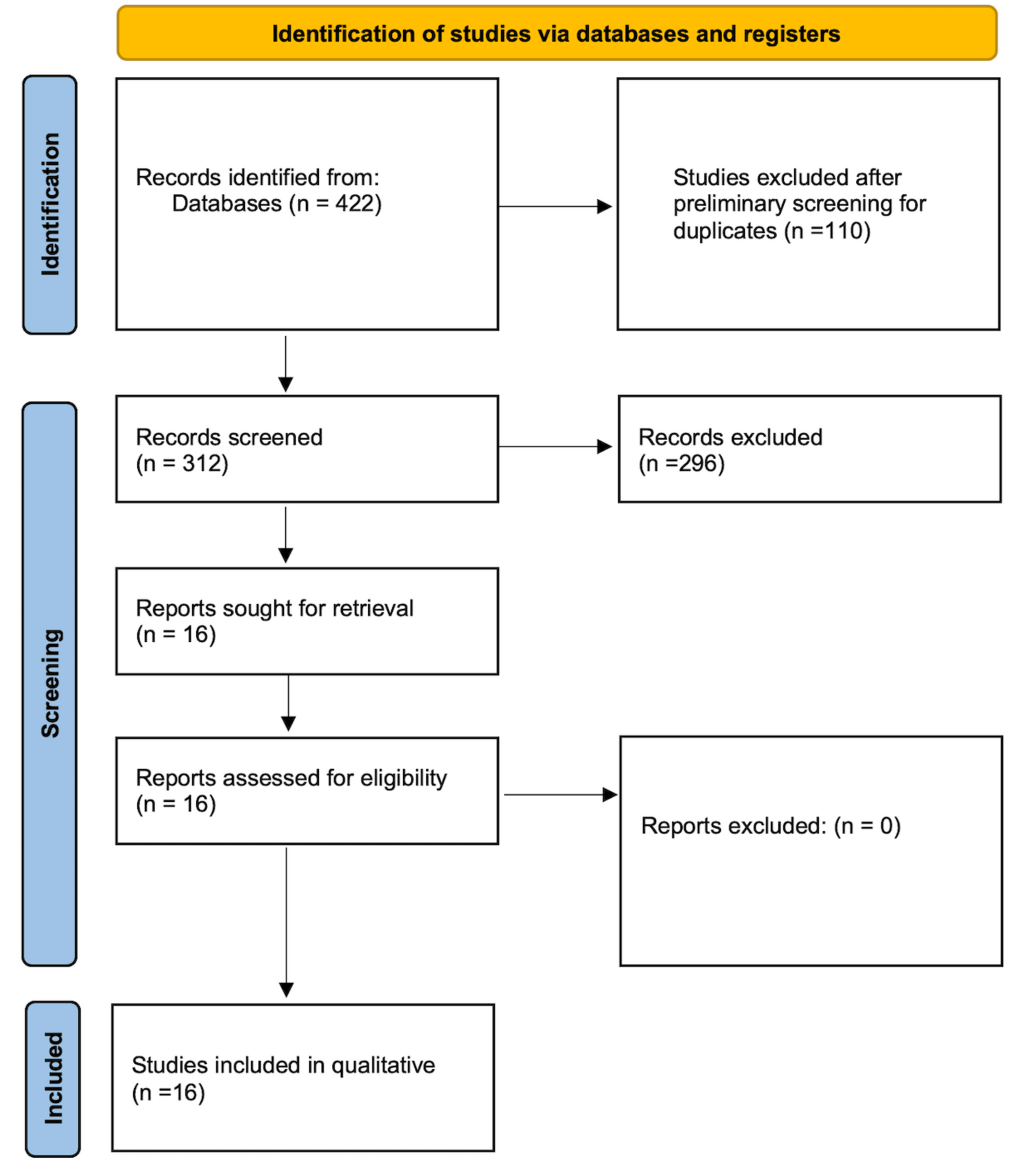
A PRISMA flow chart depicting the study selection process for this systematic review PRISMA: Preferred Reporting Items for Systematic Reviews and Meta-Analyses

The number of articles found via searching the bibliographic databases PubMed, EMBASE, and CENTRAL was 422. After removing duplicates, the number of original articles to screen was 312. Screening of the title and abstract of these articles was performed independently by two of the authors. Articles meeting criteria for further evaluation of full text numbered 16. All of these studies were included in the final review for narrative synthesis (Figure 1).

Study Characteristics

The characteristics of the studies included are detailed in Table 2. A total of 16 studies were included in this systematic review [16-31]. The included studies comprised one randomised controlled trial comparing SEMS to balloon tamponade, one prospective cohort study, nine retrospective cohort studies, one case series, and four case reports. In total, 246 patients were included, with 209 males and 37 females. The mean age of the included patients was 56. All included papers were published between 2006 and 2022. The MELD score was not reported in eight included studies [17, 18, 22, 25, 27, 29, 30, 31]. In those studies that reported the MELD score [16, 19-21, 23, 24, 26, 28], the average score was 25.

**Table 2:** Characteristics of the included studies, namely study design, year of publication, number of patients, demographic breakdown of the included patients, and the reported Model for End-Stage Liver Disease score

Study ID	Study design	Year of publication	Number of patients	Male/Female	Mean age (years)	Model for End-Stage Liver Disease Score (Mean)
Escorsell et al. [19]	Randomized controlled trial	2016	28	25/3	61.5	17
Khan et al. [16]	Retrospective Cohort Study	2022	30	20/10	53	20.3
Jain et al. [17]	Case Series	2018	3	3 males	48	Report in one patient - 35
Currais et al. [18]	Case Report	2021	1	1 male	78	Not reported
Pfisterer et al. [20]	Retrospective cohort study	2018	34	28/6	55.5	18
Fierz et al. [21]	Retrospective cohort study	2013	7	5/2	57	28
Hubmann et al. [22]	Retrospective cohort study	2006	20	18/2	52	Not reported
Wright et al. [23]	Retrospective cohort study	2010	10	9/1	49	25
Goenka et al. [24]	Retrospective cohort study	2018	12	11/1	53	20
Buechter et al. [25]	Case report	2016	1	1 female	57	Not reported
Maiwall et al. 26]	Retrospective cohort study	2017	35	34/1	46	39
Dechene et al. [27]	Case report	2009	1	1 male	59	Not reported
Fouly et al. [28]	Retrospective cohort study	2012	8	6/2	63	29
Mishin et al. [29]	Case report	2010	1	1 male	49	Not reported
Zehetner et al.[30]	Prospective cohort study	2006	39	33/6	56	Not reported
Zakaria et al. [31]	Retrospective cohort study	2013	16	14/2	56	Not reported

Risk of Bias Assessment

Table 3 depicts the risk of bias assessment tools used and the results for each study. Of the 16 included studies, one was a randomised controlled trial, which was found to have a low risk of bias [19]. Nine of the included studies were retrospective cohort studies, which were found to have a moderate risk of bias [16, 20-24, 26, 28, 31]. One study was a prospective cohort study, which had a moderate risk of bias [30]. The four case reports were all deemed high risk of bias [18, 25, 27, 29]. The single-case series was also deemed at high risk of bias [17].

**Table 3:** Outline of the risk of bias tool used to assess each study's risk of bias JBI: Joanna Briggs Institute, an international research and development organisation within the Faculty of Health Sciences at the University of Adelaide.

Study ID	Study design	Risk of bias assessment tool used	Risk of bias
Escorsell et al. [19]	Randomised controlled trial	Cochrane Collaboration's risk of bias tool	Low risk
Khan et al. [16]	Retrospective cohort study	JBI’s cohort study checklist	Moderate risk
Jain et al. [17]	Case Series	JBI’s case series checklist	High risk
Currais et al. [18]	Case Report	JBI’s case series checklist	High risk
Pfisterer et al. [20]	Retrospective cohort study	JBI’s cohort study checklist	Moderate risk
Fierz et al. [21]	Retrospective cohort study	JBI’s cohort study checklist	Moderate risk
Hubmann et al. [22]	Retrospective cohort study	JBI’s cohort study checklist	Moderate risk
Wright et al. [23]	Retrospective cohort study	JBI’s cohort study checklist	Moderate risk
Goenka et al. [24]	Retrospective cohort study	JBI’s cohort study checklist	Moderate risk
Buechter et al. [25]	Case report	JBI’s case report checklist	High risk
Maiwall et al. [26]	Retrospective cohort study	JBI’s cohort study checklist	Moderate risk
Dechene et al. [27]	Case report	JBI’s case report checklist	High risk
Fouly et al. [28]	Retrospective cohort study	JBI’s cohort study checklist	Moderate risk
Mishin et al. [29]	Case report	JBI’s case report checklist	High risk
Zehetner et al. [30]	Prospective cohort study	JBI’s cohort study checklist	Moderate risk
Zakaria et al. [31]	Retrospective cohort study	JBI’s cohort study checklist	Moderate risk

Primary Outcomes

The primary outcome for this study was six-week survival rates. Five of the included studies did not report on this outcome [17, 18, 24, 29, 31]. An average survival rate of 49% was seen among the 11 studies that reported a six-week survival rate [16, 19-23, 25-28, 30], which included a cohort of 225 patients (Table 4).

**Table 4:** Outline of each study’s reported six-week survival rate

Study ID	Number of patients	Mean age (years)	Six-week survival rate
Escorsell et al. [19]	28	61.5	54%
Khan et al. [16]	30	53	66%
Jain et al. [17]	3	48	Not reported
Currais et al. [18]	1	78	Not reported
Pfisterer et al. [20]	34	55.5	26.5%
Fierz et al. [21]	7	57	28%
Hubmann et al. [22]	20	52	80%
Wright et al. [23]	10	49	50%
Goenka et al. [24]	12	53	Not reported
Buechter et al. [25]	1	57	100%
Maiwall et al. [26]	35	46	26%
Dechene et al. [27]	1	59	0%
Fouly et al. [28]	8	63	38%
Mishin et al. [29]	1	49	Not reported
Zehetner et al. [30]	39	56	74%
Zakaria et al. [31]	16	56	Not reported

Secondary Outcomes

Control of acute bleeding: The Baveno consensus group defines the acute bleeding episode in AVB as being from the index incident (melena/haematemesis) to five days post incident. Thirteen of the 16 included studies reported on this outcome [16, 18, 20-25, 27-31]. The average rate for controlling an acute bleeding episode in patients receiving SEMS was 96% (Table 5).

**Table 5:** Each study's rate of controlling the acute bleeding episode, length of time the self-expanding metal stent was left in situ (average), and the re-bleeding rate of removal of the self-expanding metal stent (average)

Study ID	Number of patients	Mean age (years)	Rate of control of acute bleeding	Length of time stent in situ (Mean)	Re-bleeding rate on stent removal
Escorsell et al. [19]	28	61.5	This study used a modified 15-day bleeding assessment; no bleeding at 15 days in 11 stent patients (85%)	5 days	Not reported
Khan et al. [16]	30	53	87.5%	6	3%
Jain et al. [17]	3	48	Insufficient detail to report	3 days	Not reported
Currais et al. [18]	1	78	100%	11	0%
Pfisterer et al. [20]	34	55.5	79%	5 days	8.8%
Fierz et al. [21]	7	57	57%	3 days	0%
Hubmann et al. [22]	20	52	100%	6 days	0%
Wright et al. [23]	10	49	60%	9 days	0%
Goenka et al. [24]	12	53	Not reported	18 days	8%
Buechter et al [25]	1	57	100%	11 days	0%
Maiwall et al. [26]	35	46	89%	Not reported	Not reported
Dechene et al. [27]	1	59	100%	6 days	0%
Fouly et al. [28]	8	63	100%	11 days	50%
Mishin et al. [29]	1	49	100%	8 days	0%
Zehetner et al. [30]	39	56	100%	5 days	0%
Zakaria et al. [31]	16	56	88%	3 days	0%

Length of time stent in situ: Fifteen of the included studies reported on this outcome [16-25, 27-31]. The average time the SEMS was left in situ in these included studies was 7.5 days, with a maximum average duration of 18 days reported in one study [24] (Table 5).

Re-bleeding rate on removal of SEMS: Thirteen of the included studies reported on this outcome [16, 18, 20-25, 27-31]. The total number of re-bleeding episodes reported across these studies was nine. The total number of patients included in those 13 studies was 180, giving a re-bleeding rate of 5% (Table 5).

Transfusion requirement: Three of the included studies reported on this outcome [19, 27, 31]. Escorsell et al. [19], in their randomised controlled trial, reported a median requirement of two units of packed red cells for patients who received a SEMS versus six units for patients who were randomised to balloon tamponade. Dechêne et al. [27] reported no requirement for transfusion following the placement of the SEMS in their patient. Zakaria et al. [31] reported a mean of three units of packed red blood cells being required by patients post-SEMS placement in their cohort.

Bridge to further treatment: Thirteen of the included studies reported the need for further therapy [16-22, 25, 28, 30, 31]. The overall further therapy rate was 29%, with TIPSS being the most commonly performed procedure. Table 6 outlines in more detail what further therapy patients received.

**Table 6:** Breakdown of the further therapies used in patients who received a self-expanding metal stent (SEMS) TIPSS: transjugular intrahepatic portosystemic shunt

Study ID	Number of patients	Mean age (years)	Salvage/further therapy following SEMS placement
Escorsell et al. [19]	28	61.5	TIPSS in 4 patients
Khan et al. [16]	30	53	TIPSS in 12 patients
Jain et al. [17]	3	48	Nil
Currais et al. [18]	1	78	TIPSS
Pfisterer et al. [20]	34	55.5	TIPSS in 4 patients
Fierz et al. [21]	7	57	TIPSS in 3 patients
Hubmann et al. [22]	20	52	5 had TIPSS, 5 repeat banding, 5 had azygoportal disconnection
Wright et al. [23]	10	49	Not reported
Goenka et al. [24]	12	53	Not reported
Buechter et al. [25]	1	57	TIPSS
Maiwall et al. [26]	35	46	Not reported
Dechene et al. [27]	1	59	Not reported
Fouly et al. [28]	8	63	1 had TIPSS and 1 needed a liver transplant
Mishin et al. [29]	1	49	Nil
Zehetner et al. [30]	39	56	11 patients had band therapy, 8 had TIPSS
Zakaria et al. [31]	16	56	3 patients had band ligation, 7 had sclerotherapy

Adverse Events

Stent-related adverse events were reported in 11 of the included studies, with an overall rate of 25% [16, 17, 19-23, 27, 28, 30, 31]. One of the included studies did not report on adverse event rates [26]. The most common stent-related adverse event was stent migration, occurring in 36 cases. The most severe stent-related adverse event was compression of the left main bronchus, which necessitated the removal of the stent; this occurred in 2 cases. The most commonly used stent was the SX-ELLA Danis stent (Table 7).

**Table 7:** Stents used in each study alongside the adverse event rates related to it

Study	Stent brand used	Stent-related adverse event rate	Adverse event detail
Escorsell et al. [19]	SX-ELLA Danis stent	2/13 patients	1 oesophageal ulcer, 1 broncho-aspiration not causing pneumonia
Khan et al. [16]	SX-ELLA Danis stent	4/30	2 stent migrations, 2 stent-induced ulcers
Jain et al. [17]	SX-ELLA Danis stent	2/3	1 stent mispositioning requiring reposition, 1 stent migration into the stomach requiring removal
Currais et al. [18]	SX-ELLA Danis stent	0/1	Not applicable
Pfisterer et al. [20]	SX‐ELLA Danis stent	17/34	13 stent migrations, 4 oesophageal ulcerations
Fierz et al. [21]	SX-ELLA Danis stent	2/7	2 stent migrations to the stomach
Hubmann et al. [22]	Choo stent diameter of 18 mm, length of 140 mm; Ella-Boubela stent diameter of 20 mm, length of 95 mm; SX-ELLA Danis stent diameter of 25 mm, length of 135 mm)	5/20	5 stent migrations into the stomach and stent migration to the stomach were observed in five patients (one of the two patients with Choo stents, two of the three patients Ella-Boubela stents, and two of the 15 patients with SX- Ella-Danis stents)
Wright et al. [23]	SX-Ella Danis stent	2/10	2 episodes of stent failure: one where the gastric balloon failed to inflate and one where the balloon ruptured after inflating
Goenka et al. [24]	SX-Ella Danis stent	0/12	Not applicable
Buechter et al. [25]	Ella SX Danis stent	0/1	Not applicable
Maiwall et al. [26]	SX-Ella Danis stent	Not reported	Not reported
Dechene et al. [27]	SX-Ella Danis stent	1/1	Compression of the left main bronchus necessitating removal of the stent
Fouly et al. [28]	SX-Ella Danis stent	1/8	Left main bronchus obstruction requiring stent extraction
Mishin et al. [29]	SX-ELLA Danis stent	0/1	Not applicable
Zehetner et al. [30]	SX-ELLA Danis stent	7/39	7 stent migrations
Zakaria et al. [31]	SX-ELLA Danis stent	6/16	6 stent migrations

Discussion

As previously described, AVB poses many difficulties for the treating physician. The use of SEMS in its management is an area of active research. This study represents the most up-to-date and detailed review of the topic thus far.

We identified 16 suitable studies for review. These comprised one randomised controlled trial comparing SEMS to balloon tamponade, one prospective cohort study, nine retrospective cohort studies, four case reports, and one case series. In total, 246 patients were included.

Overall, this represents poor-quality data. When we consider the risk of bias assessment conducted as part of this study using validated tools, only one study was deemed low risk of bias [19]. Of the remaining 15 studies, 10 were at moderate risk of bias [16, 20-24, 26, 28, 30, 31], and five were considered high risk [17, 18, 25, 27, 29] (Table 3). However, it is essential to acknowledge that this group of patients is a challenging cohort to study, given the severity of their initial presentations and difficulty obtaining consent to be enrolled in a trial.

As we have previously stated, the Baveno consensus group has advocated using a six-week survival rate as the primary outcome when assessing the management of AVB. Five of the included studies did not report this outcome. An average survival rate of 49% was seen among the 11 studies that reported a six-week survival rate; this included a cohort of 225 patients. We propose that a six-week survival rate is an inadequate metric for assessing the management of AVB. Considering the average MELD score reported in this study was 25, this patient cohort is critically ill. Their mortality rates from all causes, not solely secondary to AVB, would undoubtedly be higher. A better assessment of techniques for managing AVB should centre around rates of controlling the acute bleeding episode, rates of re-bleeding following removal of the device, length of time the device can be left in situ, and rates at which patients are successfully bridged to more definitive treatments such as the TIPSS procedure. These parameters would indicate how successful methods, including SEMS, are in acting as a bridge to more definitive procedures.

As seen in Table 5, the SEMS were left in situ for an average of 7.5 days, with a maximum average of 18 days in one study. This has numerous advantages, such as providing time to stabilise and optimise the patient. With the stent in situ, these patients can receive enteral nutrition and do not need to be intubated, as is often required in patients using the balloon tamponade technique. Furthermore, when we assess the efficacy of the SEMS in acting as a bridge to definitive therapy (Table 6), this occurred in 29% of cases. Wright et al. [23] also make the interesting point that SEMS may act as definitive treatment in patients who are not fit or have contraindications to TIPPS.

The average rate for controlling the acute bleeding episode in patients receiving a SEMS was 96%. The added benefit of SEMS in AVB is that further endoscopic procedures can be performed to identify bleeding from other sites, such as peptic ulcers or gastric varices. This would not be possible with the balloon tamponade technique. The re-bleeding rate on the removal of SEMS was 5%, far superior to the widely reported 50% re-bleeding rate for the balloon tamponade technique.

The adverse event profile for the SEMS appears superior to the balloon tamponade technique overall. The rate of stent-related adverse events in patients receiving a SEMS was 25%. The most common adverse events were technical issues related to stent functioning, namely stent migration. The most severe stent-related adverse event was compression of the left main bronchus in two cases, which required the removal of the stent. The most commonly used stent in these studies was the SX-ELLA Danis stent, which measures 135 mm long and has an intraluminal stent diameter of 25 mm and a flange diameter of 30 mm. This results in significant radial forces. These forces are needed to achieve the haemostatic effect but have the potential to compress surrounding structures. Balloon tamponade is associated with increased risks of aspiration pneumonia, oesophageal perforation, and mucosal ischaemia, which would appear to be a more severe adverse event profile.

Our study has several limitations, which we have alluded to throughout the paper. The studies on this issue are of poor quality, with only one randomised controlled trial performed. As a result, we must interpret the results of our research with caution.

## Conclusions

Our study supports the use of SEMS in managing AVB as a promising area of research. We have highlighted that further well-designed randomised controlled trials are needed to assess the efficacy of this technique, ideally compared directly to the balloon tamponade technique. However, based on this systematic review, the current body of evidence would suggest that the SEMS is superior to the balloon tamponade technique in terms of adverse event profile, re-bleeding rate, and length of time the device can safely be left in situ. Current evidence suggests that SEMS are as effective at controlling acute bleeding episodes as the balloon tamponade technique.
